# Annual acknowledgement of manuscript reviewers

**DOI:** 10.1186/1475-925X-12-67

**Published:** 2013-07-17

**Authors:** Kenneth R Foster

**Affiliations:** 1 Department of Bioengineering, University of Pennsylvania, Philadelphia, PA, USA

## Abstract

**Contributing reviewers:**

The Editor of BioMedical Engineering Online would like to thank all the Reviewers who have contributed to the journal in Volume 11 (2012).

## 

Shouyan Wang

China

Ataollah Abbasi

Iran

Melissa Aldrich

United States of America

Clif Alferness

United States of America

Bassam Al-naami

Jordan

Tareq Alrefae

Kuwait

Kai-Nan An

United States of America

Ioannis Anagnostopoulos

Greece

Roberto Antonicelli

Italy

Ahmad Taher Azar

Egypt

Heliana B. Soares

Brazil

Katja Bähler

Austria

Rupak Banerjee

United States of America

Rafael Barea

Spain

David Barton

United Kingdom

Andres Beiras-Fernandez

Georgia

Balazs Benyo

Hungary

Joseph Bidwell

United States of America

Frank Borg

Finland

Paolo Bottoni

Italy

Paolo Brambilla

Italy

Gil Bub

United Kingdom

Julia Buján

Spain

Matthew Burfeindt

United States of America

Christopher Butson

United States of America

Giovanni Calcagnini

Italy

Victor Candia

Switzerland

Oscar Candia

United States of America

Gabriele Candiani

Italy

Ewart Carson

United Kingdom

Joao Carvalho

Brazil

Nuri Celik

United States of America

Raquel Cervigon

Spain

Rosa H M Chan

Hong Kong

Kang-Ming Chang

Taiwan

Narattaphol Charoenphandhu

Thailand

Geoff Chase

New Zealand

Mohd Zulfaezal Che Azemin

Malaysia

Yueh-Sheng Chen

Taiwan

Jyh-Cheng Chen

Taiwan

Hui Chen

United States of America

Tsung-Lin Cheng

Taiwan

Da-Chuan Cheng

Taiwan

Naomi Chesler

United States of America

Congo Tak-Shing Ching

Taiwan

Li-Shan Chou

United States of America

Eric Chua

Singapore

Liao Chun-Chih

Taiwan

Soon-Cheol Chung

Korea, South

Edward Ciaccio

United States of America

Mario Cifrek

Croatia

Maureen Clerc

France

David Cooper

Canada

Scott Cooper

United States of America

Anna Cysewska-Sobusiak

Poland

Rafael Davalos

United States of America

Daniele De Luca

Italy

Jan Casper De Munck

Netherlands

Jude DePalma

United States of America

Thomas Desaive

Belgium

Thomas Deserno

Germany

Jean-Luc Diehl

France

Jian Dong

Japan

Cyril Donnelly

Australia

Elisabetta Donzelli

Italy

Ivan Dotsinsky

Bulgaria

Barry Doyle

Australia

Andreas Drynda

Germany

Anders Eklund

Sweden

Kevin Englehart

Canada

Daniel Espino

United Kingdom

Luca Faes

Italy

Ahmed Fahmy

Egypt

Leon Farhy

United States of America

Jocelyne Fayn

France

Aaron Fenster

Canada

Giselle Ferrarri Ronque

Brazil

Aníbal Ferreira

Portugal

Anthony Fleury

France

Nils Daniel Forkert

Germany

Kenneth Foster

United States of America

Thomas Frauenfelder

Switzerland

Corinne Fredouille

France

Alessandro Frignani

Italy

Masakatsu Fujie

Japan

Miriam Furst

Israel

Marcelo Gama de Abreu

Germany

Paulo Garcia

United States of America

Lutz Gärtner

Germany

Sandrine Gerber

Switzerland

Khurshid Ghani

United Kingdom

Giovanni Giambene

Italy

Frank Gijsen

Netherlands

Robert Gilmour

United States of America

Aaron Goldstein

United States of America

He Gong

China

Federico Gordo

Spain

Peadar Grant

Ireland

Dirk Grotemeyer

Luxembourg

Josef Guttmann

Germany

Lisbet Haglund

Canada

James Hall

United States of America

Man Yong Han

Korea, South

Kamran Hassani

Iran

Hossein Hassani

United Kingdom

Gunnar Hasselgren

United States of America

Ilmo Hassinen

Finland

Fabio Hedayioglu

Portugal

Jake Heiney

United States of America

Stefan Hesse

Germany

José Antonio Hinojosa

Spain

Markus Hoenicka

Germany

Mark Holcomb

United States of America

Bart Hoogeboom

Netherlands

Tim Howells

Sweden

Cheng-Hsiung Hsieh

Taiwan

Yeh-Liang Hsu

Taiwan

Xiao Hu

United States of America

Albert Huang

United States of America

Heikki Huttunen

Finland

Hieu Huynh

Vietnam

Dimitris Iakovidis

Greece

Chang-Hwan Im

Korea, South

Gillian Johnson

New Zealand

Yasser Kadah

Egypt

Masamichi Kamihira

Japan

Ehsan Kamrani

Canada

Masa Kanduser

Slovenia

Dan Stieper Karbing

Denmark

Haruhisa Kato

Japan

Andreas Keil

United States of America

Andreas Keil

Germany

Justin Keogh

Australia

Ahsan Habib Khandoker

Australia

Joo Kim

United States of America

Mina Kim

Hong Kong

Hyunggun Kim

United States of America

Jonghwa Kim

Germany

Kio Kim

United States of America

Joonmo Kim

Korea, South

Marieke Kloosterman

Netherlands

Gary Knott

United States of America

Hans Koch

Germany

Joachim Kohn

United States of America

Terry Koo

United States of America

Cemal Köse

Turkey

Tomaz Kosmac

Slovenia

Panagiotis Krallis

Greece

Dorota Krasowska

Poland

Johann Kraus

Germany

Dejan Krizaj

Slovenia

Ching-Sung Kuo

Taiwan

Nicholas Kurniawan

Singapore

Sophie Lalande

United States of America

Philip Langley

United Kingdom

Matthias W Laschke

Germany

Chi Hieu Le

United Kingdom

Jinseok Lee

United States of America

Bart Peter Leroy

Belgium

Aaron Leung

Hong Kong

Haiyun Li

China

Lieber Li

Taiwan

Junbo Li

China

Li Li

China

Hong Lin

United States of America

Xinyang Liu

United States of America

Xin Liu

United States of America

Bindong Liu

United States of America

Men-Tzung Lo

Taiwan

Fabien Lotte

France

Edmond Lou

Canada

Cora Lueders

Germany

Miodrag Lukic

Serbia

Sai Ma

United States of America

Costis Maganaris

United Kingdom

Morteza Mahmoudi

Iran

Arthur Mak

Hong Kong

Mauro Malve

Spain

Chemseddine Mansouri

France

Haojie Mao

United States of America

Jefferson L B Marques

Brazil

Andrew Martin

United States of America

Daniel Martin

United Kingdom

Edgar Matida

Canada

Jukka Matinlinna

Hong Kong

George Matsopoulos

Greece

Connie Mayer

Canada

Owen Mays

United States of America

Sabato Mellone

Italy

Luciano Menegaldo

Brazil

Dennis Desheng Meng

United States of America

Fábio Augusto Menocci Cappabianco

Brazil

Erika Meraz

United States of America

Michael Moffitt

United States of America

Nader A. Rahman Mohamed

Egypt

Filippo Molinari

Italy

Knut Möller

Germany

Ken Monson

United States of America

Muthu Rama Krishnan Mookiah

Singapore

André Mora

Portugal

Mikhail Moshkov

Saudi Arabia

Alessio Murgia

Netherlands

Syed Murtuza Baker

Germany

Hiroshi Nakagawa

United States of America

Slawomir Nasuto

United Kingdom

Raghu Natarajan

United States of America

Xavier Navarro

Spain

Wendy Newbury

Australia

Jonathan Newell

United States of America

Masaki Nomura

Japan

Scott Nyberg

United States of America

Anders Nygren

Canada

Maxim Osipov

United Kingdom

Julien Oster

United Kingdom

Zeynep Özkurt

Turkey

Ramaswamy Palaniappan

United Kingdom

Li Pan

United States of America

Maria Papadogiorgaki

Greece

Periklis Papavasileiou

Greece

David Pasquier

France

Volkan Patoglu

Turkey

Kara Patterson

Canada

Leandro Pecchia

United Kingdom

Alessandra Pedrocchi

Italy

Mihriban Pekguleryuz

Canada

Xiongqi Peng

China

Vincenzo Penna

Germany

Susan Pfeiffer

Canada

Marie Pickerill

United States of America

James Pilla

United States of America

Jane Pillow

Australia

Ian Piper

United Kingdom

Salvatore Pirozzi

Italy

Alberto Porta

Italy

Aike Qiao

China

Kai-Rong Qin

China

Chenghu Qin

China

Hossein Rabbani

Iran

Manku Rana

United States of America

Sohan Ranjan

India

Lei Ren

United Kingdom

Thomas Riedel

Switzerland

Alejandro Riera

Spain

Marcelo Risk

Argentina

Pere Riu

Spain

M Carmen Romano

United Kingdom

Daniel Romero

Spain

François Rousseau

France

Emilio Sacristan

Mexico

Leyla Sadighpour

Iran

Tolga Atilla Sagban

Germany

Mikel Sagues

Spain

Arash Salarian

Switzerland

Solomon Samuel

United States of America

Nejc Sarabon

Slovenia

Michalis Savelonas

United States of America

Lukas Schaupp

Austria

Gerd Schmalisch

Germany

Jon Schwartz

United States of America

Christopher Seal

New Zealand

Alexander Seifalian

United Kingdom

Frederic Senny

Belgium

Babak Seyed-Aghazadeh

United States of America

Danial Shahmirzadi

United States of America

Baoci Shan

China

Qi Shao

United States of America

Lingling ShuiLinglingS

China

Riyanto Sigit

Indonesia

David Simpson

United Kingdom

Ramesh Singh

Malaysia

Rakesh Sinha

India

Arkadiusz Sitek

United States of America

Bjørn Skallerud

Norway

John Skedros

United States of America

Ni Ni Soe

Singapore

Dimitrios Sokolis

Greece

Manuchehr Soleimani

United Kingdom

William Song

United States of America

Kriangkrai Sooksood

Germany

Iis Sopyan

Malaysia

Abdullah Ruhi Soylu

Turkey

Lambert Speelman

Netherlands

Paul Steendijk

Netherlands

Irma Sterpi

Italy

Fong-Chin Su

Taiwan

Yankui Sun

China

Raji Sundararajan

United States of America

Ashwath Sundaresan

New Zealand

Cathryn Sundback

United States of America

Kyunghyun Sung

United States of America

Hak-Joon Sung

United States of America

Hiroki Tamura

Japan

Masanori Tamura

Japan

Liwen Tan

China

Simon Tang

United States of America

David Thomas

Australia

Ryo Torii

United Kingdom

Beatriz Trenor

Spain

Khoa Troung Quang Dang

Vietnam

Markos Tsipouras

Greece

Alexander Tsouknidas

Greece

Yuan-Kun Tu

Taiwan

Ali Bulent Usakli

Turkey

Marcel van 't Veer

Netherlands

Paritosh Vasava

Finland

Karthik Vishwanath

United States of America

Marc Vuylsteke

Belgium

Jessica Wagenseil

United States of America

Alvin Wald

United States of America

Baikun Wan

China

Pengfei Wang

Ireland

John Wang

United States of America

Jinwu Wang

China

Tao Wang

United States of America

Weidong Wang

China

Qing Wang

China

Junchen Wang

China

Po-Shan Wang

Taiwan

Jia-Jung Wang

Taiwan

Betsy Waterman

United States of America

Richard Weir

United States of America

Tadeusz Wilczok

Poland

Malgorzata E Wilinska

United Kingdom

Caroline Wilkinson

United Kingdom

Jeremy Windsor

United Kingdom

Michael Woeltje

Germany

Wei Wu

United States of America

Yunfeng Wu

China

Shyi-Kuen Wu

Taiwan

Wenlan Wu

Taiwan

Qi Xu

China

Sheng Yan

China

Lang Yang

United Kingdom

Xiaofeng Yang

United States of America

Chia-Yen Yang

Taiwan

Jong Chul Ye

Korea, South

Gregory Yourek

United States of America

Nasser Yousif

United States of America

Silvano Zanutto

Argentina

Rad Zdero

Canada

Guangping Zeng

China

Zijing Zeng

United States of America

Yongjun Zhai

United States of America

Hui Zhang

China

Shaohong Zhang

Hong Kong

Zhanqi Zhao

Germany

Tianyu Zhao

United States of America

Heng Zhao

United States of America

Guoyan Zheng

Switzerland

Changyu Zheng

United States of America

